# Measuring sociocultural engagement of health professions education students: a psychometric study

**DOI:** 10.1186/s12909-024-05566-0

**Published:** 2024-05-23

**Authors:** Salah Eldin Kassab, Ramya Rathan, Henk G. Schmidt, Hossam Hamdy

**Affiliations:** 1 College of Medicine, Gulf Medical University, Ajman, United Arab Emirates; 2 Department of Physiology, Faculty of Medicine, Suez Canal University, Ismailia, Egypt; 3 Institute of Medical Education Research, Erasmus MC, Rotterdam, The Netherlands; 4 Department of Psychology, Education and Child Studies, Erasmus University Rotterdam, Rotterdam, The Netherlands

**Keywords:** Student engagement, Sociocultural engagement, Validity

## Abstract

**Background:**

Sociocultural engagement of students refers to broadening viewpoints and providing awareness of, and respect for, diverse backgrounds and perspectives. However, there are no existing validated instruments in the literature for measuring sociocultural engagement of health professions education (HPE) students. Therefore, the aim of this study is to develop and validate a questionnaire designed to assess sociocultural engagement among HPE students.

**Methods:**

The study included undergraduate HPE students (n = 683) at Gulf Medical University. The initial version of the sociocultural engagement of students’ questionnaire (SESQ) was developed after extensive literature review and guided by the Global Learning Qualifications Framework. We then tested the content validity of the questionnaire by using focus group discussion with subject experts (n = 16) and pilot testing with students (n = 20). We distributed the content-validated version of the SESQ (16 items) to undergraduate students in six HPE colleges. To examine the construct validity and construct reliability of the questionnaire, we conducted exploratory factor analysis, followed by confirmatory factor analysis.

**Results:**

Confirmatory factor analysis supported the two-factor structure which consists of 13 items with good fitness indices (χ^2^ = 214.35, df = 61, χ ^2^/df = 3.51, CFI = 0.98, RMSEA = 0.06, SRMR = 0.025, and AIC = 208.00). The two factors were sociocultural interactions (8 items) and sociocultural adaptation (5 items). The construct reliability of the total questionnaire is 0.97 and the two factors were 0.93 and 0.92 for sociocultural interactions and sociocultural adaptation, respectively. In addition, there were significant weak correlations between both factors of sociocultural engagement scores and student satisfaction with the university experience (*r* = .19 for each, *P* = .01).

**Conclusions:**

The sociocultural engagement of students’ questionnaire exhibits good evidence of construct validity and reliability. Further studies will be required to test the validity of this questionnaire in other contexts.

**Supplementary Information:**

The online version contains supplementary material available at 10.1186/s12909-024-05566-0.

## Introduction

In an era of growing globalization, heightened international migration, and increased cultural diversity within nations, there is a need to progress the development of research on sociocultural engagement of health professionals. The higher education accrediting bodies stipulate that health professions students should demonstrate the skills of social and cultural competence [1, 2]. Success in a clinical encounter requires good understanding of the sociocultural dimensions that shape a patient’s health values, beliefs, and behaviors. Proficiency in this competency is imperative for ensuring equitable and effective healthcare delivery to patients from diverse backgrounds. Sociocultural engagement, as outlined in the Global Learning Qualifications Framework (GLQF), involves broadening perspectives and fostering an understanding of, and respect for, various backgrounds and viewpoints [3]. Numerous research studies have examined the multifaceted aspects of student engagement, encompassing cognitive, behavioral, and emotional dimensions [4, 5]. Nonetheless, within the field of health professions education (HPE), there exists a notable gap in the exploration of the sociocultural dimension of student engagement.

According to Wenger’s theory, learning is conceptualized as social participation, encompassing active engagement in activities alongside individuals and practices within community settings [6]. Furthermore, it involves the construction of identities within these social communities, and the cognitive processes of meaning-making and interpretation associated with the activities undertaken within these contexts [6, 7]. Sociocultural engagement develops when students embed themselves in a new social group and develop their individual identity. The development of student identity reduces the gap between their own social and cultural values and the new community. The social aspect of student engagement is rooted in the concept of academic and social integration of students with the campus. Social integration includes the student’s perceptions of the quality of interactions with peers, faculty, and staff within the institution, in addition to their participation in extracurricular activities [8, 9].

We have recently reviewed the literature and developed a comprehensive framework of student engagement illustrating the antecedents, mediators, dimensions, outcomes, and measurement of student engagement in health professions education [4, 5, 10]. The framework views engagement as an internal psychological state which is influenced by student attributes, university structures, psychosocial factors, and broader socio-cultural contexts [11, 12]. Although this framework provided a guiding structure for implementation and research of student engagement in HPE, developing an instrument for measuring this construct in an HPE context is an area of educational research that requires exploration.

Therefore, the current study is testing the practical application of this framework in relation to sociocultural engagement of HPE students. The results from this study have the potential of not only adding to the HPE literature in this under-researched area, but also as feedback into the university programs for the purpose of improving the quality of education in these programs. Accordingly, the primary aim of this study is to examine the development of the Sociocultural Engagement of Students’ Questionnaire (SESQ) in HPE and to assess the sources of evidence that support its validity and reliability.

## Methods

### Study context

This study is conducted at Gulf Medical University (GMU) in Ajman, UAE. The University consists of six constituent colleges: Medicine, Dentistry, Pharmacy, Health Sciences, Nursing, and Healthcare Management and Economics. The six colleges are in one campus with sharing of physical and human resources in the academic activities of the university. The university is a mix of students from multiple cultural and social backgrounds with students representing 77 nationalities. The university fosters a collaborative learning environment where students from different healthcare disciplines come together to acquire a comprehensive understanding of teamwork and communication in the healthcare domain. Therefore, sociocultural engagement of students from these diverse cultures and disciplines is important for facilitating the interactions between students and shared learning.

### Development of the study instrument

The developmental process of the study questionnaire started with an extensive review of pertinent literature and was informed by our recently formulated framework describing student engagement in Health Professions Education [10]. The “*Communities of Practice*” theory served as the umbrella for explaining the sociocultural engagement of HPE students. The definition of sociocultural engagement was adapted from the Global Learning Qualifications Framework (GLQF), which was designed to facilitate the documentation and evaluation of university-level learning, with sociocultural engagement representing one of the eight learning domains identified therein [3].

Given the absence of existing instruments tailored specifically to measure the sociocultural engagement of HPE students, an initial pool of 15 questionnaire items was adapted from the questions and examples of Sociocultural and Civic Engagement of the GLQF and the items were contextualized to the HPE setting [3]. In addition, four items were adapted from the National Survey of Student Engagement (NSSE) indicator ‘*Discussion with Diverse Others*” [13].

### Sources of evidence which support the validity of the questionnaire

1. *Content validity evidence*: Content validity of the questionnaire items was ensured through a rigorous process. A focus group discussion involving medical education experts (n = 16) was conducted to ascertain the representation of the construct adequately. The participants comprised faculty members possessing postgraduate qualifications, specifically Masters or Diplomas, in the field of health professions education. Moreover, to acquaint the group with the construct under examination, the focus group session commenced with a presentation by the primary author on sociocultural engagement, drawing from existing literature to foster a shared comprehension among participants. Subsequently, the questionnaire was distributed to participants to assess the alignment of items with the intended construct, propose additional items deemed necessary for comprehensive coverage, and suggest modifications in the items to enhance clarity. The final iteration of the questionnaire was determined based on the content validity index and qualitative feedback from expert judges, after agreement by all authors.

Expert judges scored each item of the SESQ based on the criteria of “relevance,” to the measured construct and “clarity.” Each item was assessed using a three-point Likert scale for relevance (1 – not relevant, 2 – rather relevant, 3 – relevant) and “clarity” (1 – not clear, 2 – relatively clear, and 3 – clear). We examined the content validity evidence by computing Content Validity Index (CVI) using ratings of items by the expert judges. The formula for calculating CVI is the number of all the respondents in the “relevance” and “clarity” criteria divided by the number of experts who have score 3 in the relevant question in that criterion [14]. In this formula, if an item has a score of more than 0.79, that item is retained in the questionnaire. If CVI is between 0.70 and 0.79, the item is questionable and needs correction and revision, and if it is less than.70, the item is unacceptable, and it must be deleted. In addition, the experts were asked to provide suggestions for adding new items deemed relevant for measuring the construct or modifying the existing items. Furthermore, pilot testing was done with HPE students (n = 20) for testing the clarity and suitability of the questionnaire items. The pilot-tested questionnaire was then distributed through the form of google sheet. A QR Code was generated, and the code was shared with all the students. A convenience sampling was used for data collection.

2. *Internal structure evidence of validity*: Given the absence of predetermined specifications regarding the dimensionality of the sociocultural engagement construct, an Exploratory Factor Analysis (EFA) was undertaken on the 16-item questionnaire utilizing a cohort comprising 327 students. The analysis aimed to identify underlying factors and assess the structure of sociocultural engagement. Principal Component Analysis (PCA) was used with Varimax rotation. Data was examined for factor loadings and communalities to determine the significance of each item and the presence of distinct factors. Results were interpreted to understand the dimensions of sociocultural engagement among the student population.

Guided by the results of EFA, the construct validity of the developed sociocultural engagement of students’ questionnaire (SESQ) was evaluated using Confirmatory Factor Analysis (CFA) to examine the degree of fitness between the measurement model and the theoretical model. The CFA analysis was conducted on a sample of 683 HPE students. Different indices were used to assess the goodness-of-fitness of the model as we previously reported [15]. The fitness indices include Comparative Fit Index (CFI), *Chi-Square* (χ^2^) and χ^2^ / df, Root Mean Square Error of Approximation (RMSEA), Standardized Root Mean Square Residual (SRMR), and Aikaike Information Criterion (AIC) [16, 17]. A decision on what represents the best model fit takes these different indicators into account [16, 17].

3. *Predictive validity*: To examine the predictive validity of the SESQ, we tested the correlations between the sociocultural engagement scores and the student satisfaction with the university experience using *Pearson’s correlation*.

$$CR\, = \,\frac{{{{\left( {\sum {{\lambda _i}} } \right)}^2}}}{{{{\left( {\sum {{\lambda _i}} } \right)}^2} + \left( {\sum {{{{\epsilon}}_i}} } \right)}}$$

*Construct reliability (CR)*: Composite (or construct) reliability is a measure of internal consistency in the observed indicators that load on a latent variable (construct). The formula for calculating construct reliability is:

whereby, λ (lambda) is the standardized factor loading for item *I*, and ε is the respective error variance for item *i*. The error variance (ε) is estimated based on the value of the standardized loading (λ) [18].

Quantitative data are entered and analyzed using the *Statistical Package for Social Sciences (SPSS*) version 28.0. Analysis of Moment Structures (*AMOS*) program version 21 is used for studies involving confirmatory factor analysis. A *P-value* < 0.05 is considered statistically significant.

## Results

### Demographic variables

As shown in Table 1, the gender distribution of the students in the study was 73.1% and 26.9% for females and males, respectively. Almost half of the students are Asians (48.3%) while Middle Eastern students represented 39.7%. The remaining students were Africans (8.7%) and Western (3.2%). The largest percentage of participants were undergraduate medical students (58.6%). The remaining students were studying dentistry (18.2%), nursing (7%), healthcare management and economics (6.7%), pharmacy (4.8%), and health sciences (4.7%).

**Table 1:** Demographic variables in the study

	N	%
**Gender**		
Female	497	73.1
Male	183	26.9
*Sum*	*680*	
**Geographic origin**		
Asian	327	48.3
Middle Eastern	269	39.7
African	59	8.7
Western	22	3.2
*Sum*	*677*	
**Study program**		
Medicine	400	58.6
Dentistry	124	18.2
Nursing	48	7.0
Healthcare management & economics	46	6.7
Pharmacy	33	4.8
Health Sciences	32	4.7
*Sum*	*683*	

### Sources of evidence which support the validity of the sociocultural engagement of students’ questionnaire (SESQ) in HPE

#### Content validity evidence

Following comprehensive literature review, the initial SESQ discussed in the focus group with expert judges consisted of 15 items. The average CVI of the questionnaire for relevance and clarity was 0.83 and three items had extensive revision because of low CVI. An additional item was suggested by the expert judges and the final questionnaire consisted of 16 items. In addition, pilot testing with students (n = 20) resulted in minor edits in the items mainly for clarity and simple understanding.

#### Validity evidence for the internal structure

The results of EFA indicate a high level of sampling adequacy as evidenced by the Kaiser-Meyer-Olkin Measure of 0.958. Additionally, Bartlett’s Test of Sphericity revealed a significant Chi-Square statistic of approximately 3897.77 with 120 degrees of freedom (*P* < .001), suggesting that correlations among variables were sufficiently different from zero to proceed with the factor analysis. The EFA revealed the presence of two distinct components which explained 62% of the total variance. By examining the rotated matrix, the two factors were subsequently labeled as “sociocultural interactions” (comprising 8 items) and “sociocultural adaptation” (comprising 8 items).

**Fig. 1:**
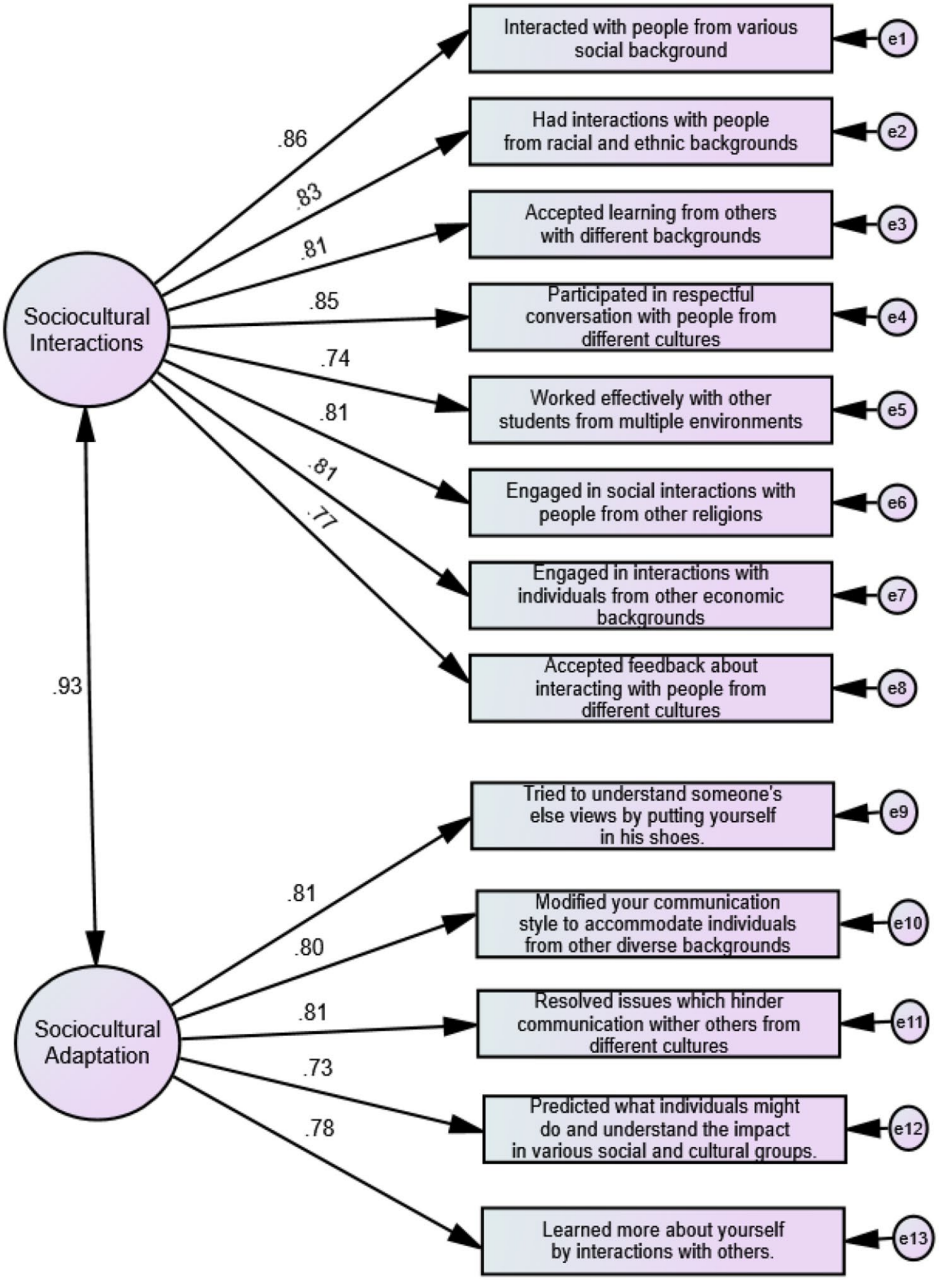
Confirmatory factor analysis of sociocultural engagement of students’ questionnaire (SESQ) in health professions education (n = 683)

As a follow-up, we subjected the data from 683 students to CFA, but as shown in Table 2, the 16-item questionnaire did not show acceptable fitness indices (χ^2^ = 808.51, df = 104, χ ^2^/df = 7.77, CFI = 0.91, RMSEA = 0.10, SRMR = 0.04, and AIC = 304.00). In contrast, the two-factor model with sociocultural interactions (8 items) and sociocultural adaptation (8 items) demonstrated better fitness. After dropping three items because of low factor loading and after using the modification indices, the two-factor model with sociocultural interactions (8 items) and sociocultural adaptation (5 items) emerged as the most parsimonious model, characterized by favorable fit indices (χ^2^ = 214.35, df = 61, χ ^2^/df = 3.51, CFI = 0.98, RMSEA = 0.06 (0.05 − 0.07), SRMR = 0.025, and AIC = 208.00). The dropped three items were: (a) *Actively sought to understand cultural norms and practices that differ from your own*., (b) *Connected your learning in the University to problems and issues in the society*, and (c) *Included diverse points of view (political, religious, racial/ethnic, gender, etc.) in course discussions or assignments*. We conducted further analyses using the 13-item model since it was the most parsimonious. Figure 1 shows factor loadings for each of the items on the latent factor with factor loadings ranging from 0.73 to 0.86. The final version of the questionnaire is available in the appendices.

**Table 2:** Model fit for measurement models of the sociocultural engagement of students questionnaire (SESQ) in health professions education (n = 683)

Study scales	χ ^2^	df	χ ^2^/ df	CFI	RMSEA (90% C.I.)	SRMR	AIC
One latent factor − 16 items	808.51	104	7.77	0.92	0.10 (0.09 − 0.11)	0.043	304.00
Two latent factors − 16 items	557.31	103	5.41	0.95	0.08 (0.07 − 0.09)	0.037	304.00
Two latent factors − 13 items	214.35	61	3.51	0.98	0.06 (0.05 − 0.07)	0.025	208.00

#### Predictive validity

The results demonstrate that sociocultural engagement weakly correlated with student satisfaction with the program experience (*r* = .19). Similarly, both sociocultural interactions and sociocultural adaptation weakly demonstrated weak correlation with student satisfaction (0.19 for each).

### Construct reliability of the SESQ

The 13-item questionnaire demonstrated excellent construct reliability (0.97). In addition, the construct reliability of the sociocultural interaction and sociocultural adaptation constructs were 0.93 and 0.92, respectively.

## Discussion

We have demonstrated in this study that the sociocultural engagement of student questionnaire (SESQ) exhibits strong evidence of construct validity and reliability. Confirmatory factor analysis demonstrated sociocultural interactions and sociocultural adaptation as two strongly interrelated constructs of sociocultural engagement. In addition, student satisfaction with the program experience is weakly correlated with the sociocultural engagement of students. Lastly, the sociocultural engagement questionnaire exhibited excellent construct reliability.

To our knowledge, this is the first study to develop and validate a questionnaire for measuring sociocultural engagement of HPE students. We demonstrated using a large sample of HPE students that the sociocultural engagement is two dimensional with two latent constructs representing the sociocultural interactions and adaptation. Other studies have examined the validity of instruments measuring conceptually related constructs such as sociocultural competence survey [19], sociocultural adaptation questionnaire [20, 21], and sociocultural questionnaire [22]. However, the sociocultural competence scale have identified three dimensions: cognitive, social, and political [19]. On the other hand, the sociocultural adaptation scale showed another three dimensions: diversity approach, social functioning, and distance and life changes [21]. In addition, studies have examined the cultural component of the construct [23], or the social engagement [24]. The comparisons across these studies, as well as their alignment with the present study, is difficult due to disparities in the conceptualization of the construct, incongruities in the sampled populations exposed to the measurement instrument, differences in the study context, and the heterogeneous nature of the content and number of items in each instrument. Furthermore, none of these studies specifically addressed the sociocultural engagement construct.

The sociocultural engagement is guided by Wenger’s theory of the community of practice (CoP). Wenger posits that students who are engaged in sociocultural activities share the feeling of community, learning by doing, learning by experiencing, and shaping their identities within social communities, a process facilitated by negotiation and interpretation in various contextual interactions [6]. The feeling of belonging develops when students construct their identity and the sense of connection to the university community [25]. In a university setting, sociocultural engagement of students occurs within the university social milieu, including social spaces, clubs and societies, the students’ union, and student accommodation [26]. Facilitating interpersonal interactions among medical students, thereby fostering social companionship mitigates their stress levels [27].

The current study demonstrated that students who perceive better sociocultural engagement had more satisfaction with their university experience. The student satisfaction with university experience has been directly linked with student retention [26]. Students who think about leaving the university are less satisfied with their university experience and appear to be less engaged with their peers and their institution [26]. Although the correlations between sociocultural engagement and student satisfaction were considered weak, this finding adds another supporting evidence for the predictive validity of the study questionnaire. However, it is essential to acknowledge that the magnitude of correlation is contingent upon the sample size employed in the study. Consequently, while weak yet statistically significant correlations may imply some level of association between the sociocultural engagement and student satisfaction, the utilization of a large sample in our study has the propensity to amplify small relationships, rendering them statistically significant. Moreover, the incorporation of additional variables in the statistical analysis may not attain statistical significance.

This study is of potential benefit for conducting empirical research using the SESQ to observe the development of the sociocultural engagement competencies of HPE students. In addition, the study has direct implication on testing the impact of institutional strategies designed to improve the sociocultural engagement and student satisfaction with HPE program experience. The university must play an important role in providing students with the opportunity to develop their social life and to practice social integration as they learn and grow [28]. Students should utilize their sociocultural communication skills to actively participate in professional and social activities, thereby addressing the power dynamics that arise during these engagements.

Although this study has many strengths, there are certain limitations that need to be reported. The cross-sectional survey-based nature of the study does not allow us to conclude cause and effect relationships between the study variables. Another inherent weakness of using questionnaires is biases in student responses and measuring self-perceptions rather than their actual engagement, sense of belonging, or university support. Finally, the context of the study being implemented in one university setting does not allow the generalization of the study findings to other educational contexts.

## Conclusions

This study described the development of a questionnaire for measuring sociocultural engagement of health professions education students. We demonstrated also different sources of evidence to support the construct validity of the questionnaire. First, the content-related evidence is supported by the evaluation of the items by medical education experts along with pilot testing with medical students. Second, validity evidence for internal structure is supported by the findings from confirmatory factor analysis demonstrating good fit between the two-dimensional model and the theoretical model. Third, predictive validity evidence is supported by the positive (though weak) correlation between sociocultural engagement and student satisfaction with the university experience. In addition, the study demonstrated excellent construct reliability of the sociocultural engagement questionnaire. The findings of this study carry implications for enhancing the sociocultural engagement of health professions education students and consequently improving the overall educational experience of HPE students.

### Electronic supplementary material

Below is the link to the electronic supplementary material.

Supplementary Material 1

## Data Availability

The datasets used and/or analysed during the current study are available from the corresponding author on reasonable request.

## References

[1] Betancourt JR (2003). Cross-cultural Medical Education: conceptual approaches and frameworks for evaluation. Acad Med.

[2] LCME. Functions and Structure of a Medical School2023; 2023(12/25/2023). https://lcme.org/publications/.

[3] Framework. Sociocultural and civic engagement. Saratoga Springs, NY: Suny Empire State College 2022 [ https://www.esc.edu/global-learning-qualifications-framework/learning-domains/engagement/.

[4] Kassab SE, El-Sayed W, Hamdy H (2022). Student engagement in undergraduate medical education: a scoping review. Med Educ.

[5] Kassab SE, Al-Eraky M, El-Sayed W, Hamdy H, Schmidt H (2023). Measurement of student engagement in health professions education: a review of literature. BMC Med Educ.

[6] Wenger E, Blackmore C (2010). Communities of practice and social learning systems: the career of a concept. Social learning systems and communities of practice.

[7] Masika R, Jones J (2015). Building student belonging and engagement: insights into higher education students’ experiences of participating and learning together. Teach High Educ.

[8] Tinto V (1993). Leaving college: rethinking the causes and cures of student attrition.

[9] Rimm-Kaufman SE, Baroody AE, Larsen RAA, Curby TW, Abry T (2015). To what extent do teacher–student interaction quality and student gender contribute to fifth graders’ engagement in mathematics learning?. J Educ Psychol.

[10] Kassab SE, Taylor D, Hamdy H. Student engagement in health professions education: AMEE Guide 152. Med Teach. 2022:1–17. 10.1080/0142159X.2022.2137018 36306374

[11] Kahu ER, Nelson K (2017). Student engagement in the educational interface: understanding the mechanisms of student success. High Educ Res Dev.

[12] Kahu ER (2013). Framing student engagement in higher education. Stud High Educ.

[13] LaNasa SM, Cabrera AF, Trangsrud H (2009). The Construct Validity of Student Engagement: a confirmatory factor analysis Approach. Res High Educt.

[14] Wilson FR, Pan W, Schumsky DA (2017). Recalculation of the critical values for Lawshe’s content validity ratio. Meas Evaluation Couns Dev.

[15] Kassab SE, El-Baz A, Hassan N, Hamdy H, Mamede S, Schmidt HG (2023). Construct validity of a questionnaire for measuring student engagement in problem-based learning tutorials. BMC Med Educ.

[16] Lt H, Bentler PM (1999). Cutoff criteria for fit indexes in covariance structure analysis: conventional criteria versus new alternatives. Struct Equation Modeling: Multidisciplinary J.

[17] Boateng GO, Neilands TB, Frongillo EA, Melgar-Quinonez HR, Young SL (2018). Best practices for developing and Validating Scales for Health, Social, and behavioral research: a primer. Front Public Health.

[18] Raykov T (2016). Estimation of Composite Reliability for Congeneric measures. Appl Psychol Meas.

[19] Hopkins M, Kurowska-Pysz J, Nowak-Zolty E, Szyszka M (2023). Attending to cross-border sociocultural competence in bilingual programs in the polish-czech border region: an exploratory study. PLoS ONE.

[20] Wilson J, Ward C, Fetvadjiev VH, Bethel A (2017). Measuring Cultural competencies: the Development and Validation of a revised measure of Sociocultural Adaptation. J Cross-Cult Psychol.

[21] Valenti GD, Magnano P, Faraci P (2022). Evaluating the dimensionality of the Sociocultural Adaptation Scale in a sample of International Students Sojourning in Los Angeles: which difference between Eastern and Western Culture?. Eur J Investig Health Psychol Educ.

[22] Hardin K, Hernandez RS, Shin TM, Ortega P. Medical student perceptions of Sociocultural issues in Healthcare: a Multisite Study of Medical Spanish Education. Teach Learn Med. 2023:1–12. 10.1080/10401334.2023.2230187 37403289

[23] Osmancevic S, Grossschadl F, Lohrmann C (2023). Cultural competence among nursing students and nurses working in acute care settings: a cross-sectional study. BMC Health Serv Res.

[24] Joshi DR, Adhikari KP, Khanal B, Khadka J, Belbase S (2022). Behavioral, cognitive, emotional and social engagement in mathematics learning during COVID-19 pandemic. PLoS ONE.

[25] Burkhardt MS, Gower S, Flavell H, Taplin J (2015). Engagement and Creation of Professional Identity in undergraduate nursing students: a convention-style orientation event. J Nurs Educ.

[26] Thomas L (2012). Building student engagement and belonging in Higher Education at a time of change: final report from the what works?.

[27] Ziegelstein RC (2018). Creating Structured opportunities for Social Engagement to promote well-being and avoid burnout in medical students and residents. Acad Med.

[28] Denson N, Zhang S (2010). The impact of student experiences with diversity on developing graduate attributes. Stud High Educ.

