# Supplementary material for: Measuring sociocultural engagement of health professions education students: a psychometric study
Source: BMC Med Educ. 2024 May 23;24:567. doi: 10.1186/s12909-024-05566-0 (PMC11119711; doi:10.1186/s12909-024-05566-0)
Supplement: Supplementary file 1 — Supplementary Material 1 [file 12909_2024_5566_MOESM1_ESM.docx]

**Student Informed Consent**

**Dear student**

The questionnaires below examine the sociocultural engagement of health professions education students.

Your input and opinions are most valuable in attaining accurate results for this study, and in turn will pave the way for improvement of the quality of education at GMU. Your individual responses will be kept confidential to the research team and will not be released to the university or to anyone else. So, please take time to complete this questionnaire honestly and openly.

In accordance with the Data Protection Act, we ask you to sign the following declaration. You can be confident that the information we collect from you will be used only for the purposes of research, and that no student will be identified in any report of our findings. Your individual responses will be kept confidential to the research team; they will not be released to the University or to anyone else. You have the right to refuse participation without any negative consequences.’

*I agree that the data collected from me may be held and processed by the team for the purpose of research.*

| Signature |  |
| --- | --- |

**Thank you**

**Background information**

Name/ID

| **Age:** |  |
| --- | --- |

**What is your gender?**

Male

Female

**Which of the following best describes your cultural background? (Select all that apply)**

African

Asian

Western

Middle Eastern

Other (please specify): __________________________

**What is your study college/program at GMU?**

Medicine

Dentistry

Pharmacy

Health Sciences

Healthcare Management

Nursing

**What is your study level at GMU?**

Year 1

Year 2

Year 3

Year 4

Year 5

**What is your latest qualification?**

High School

 Bachelor

 Master

 Other (Please specify)

**How often have you done the following in this academic year in the university?**

|  | **Item** | **Never** | **Sometimes** | **Often** | **Always** |
| --- | --- | --- | --- | --- | --- |
| 1 | Worked effectively with other students and groups in multiple environments. |  |  |  |  |
| 2 | Accepted learning from other students with diverse points of view and backgrounds. |  |  |  |  |
| 3 | Accepted feedback from other students about how you interact with people from diverse cultures. |  |  |  |  |
| 4 | Predicted what students might do and understand the impact of their actions in various social and cultural groups. |  |  |  |  |
| 5 | Participated in respectful conversation with students from different cultures who have different viewpoints |  |  |  |  |
| 6 | Had social interactions with students from racial and ethnic backgrounds other than your own |  |  |  |  |
| 7 | Interacted with students from various social backgrounds other than your own |  |  |  |  |
| 8 | Engaged in social interactions with students from religious beliefs other than your own |  |  |  |  |
| 9 | Engaged in social interactions with students from different economic levels other than your own |  |  |  |  |
| 10 | Modified your communication style to accommodate students from diverse backgrounds. |  |  |  |  |
| 11 | Learned more about yourself by interactions with other students. |  |  |  |  |
| 12 | Tried to better understand someone else's views by putting yourself in their shoes and seeing an issue from their point of view. |  |  |  |  |
| 13 | Resolved issues which hindered your communication with other students from different cultural backgrounds |  |  |  |  |

**Thank you.**
